# Foliar Application of Silicon Influences the Physiological and Epigenetic Responses of Wheat Grown Under Salt Stress

**DOI:** 10.3390/ijms252413297

**Published:** 2024-12-11

**Authors:** Renata Tobiasz-Salach, Barbara Stadnik, Marzena Mazurek, Jan Buczek, Danuta Leszczyńska

**Affiliations:** 1 Department of Crop Production, University of Rzeszow, Zelwerowicza 4 St., 35-601 Rzeszow, Poland; barbarast@dokt.ur.edu.pl (B.S.); jbuczek@ur.edu.pl (J.B.); 2 Doctoral School of the University of Rzeszow, University of Rzeszow, Rejtana 16C St., 35-959 Rzeszow, Poland; 3 Department of Physiology and Plant Biotechnology, University of Rzeszow, Ćwiklińskiej 2 St., 35-601 Rzeszow, Poland; mmazurek@ur.edu.pl; 4 Department of Crops and Yield Quality, Institute of Soil Science and Plant Cultivation—State Research Institute, Czartoryskich 8 St., 24-100 Puławy, Poland; leszcz@iung.pulawy.pl

**Keywords:** chlorophyll fluorescence, DNA methylation, gas exchange, salinity, *Triticum aestivum* L.

## Abstract

Soil salinity is considered a serious problem that limits agricultural productivity. Currently, solutions are being sought to mitigate the negative impact of salt on economically important crops. The aim of the study was to evaluate the effect of foliar application of silicon (Si) on the physiological and epigenetic responses of wheat grown under salt stress conditions. The experiment with wheat seedlings was established in pots with 200 mM NaCl added. After 7 days, foliar fertilizer (200 g L^−1^ SiO_2_) was used at concentrations of 0.05, 0.1 and 0.2%. Physiological parameters were measured three times. The addition of salt caused a significant decrease in the values of the measured parameters in plants of all variants. In plants sprayed with Si fertilizer under salinity conditions, a significant increase in CCI and selected gas exchange parameters (*P*_N_, *C*_i_, *E*, *g*_s_) and chlorophyll fluorescence (PI, RC/ABS, F_V_/F_m_, Fv/F_0_) was observed. Si doses of 0.1 and 0.2% showed a better mitigating effect compared to the dose of 0.05%. The observed effect was maintained over time. The results obtained indicate a positive role for foliar silicon fertilization in mitigating salinity stress in wheat. Epigenetic mechanisms play an important role in regulating gene expression in response to stress. Changes in the status of methylation of the 5′CCGG3′ sequence of the nuclear genome of wheat plants exposed to salinity and treated with Si at different doses were determined by the MSAP approach. The obtained results showed a clear alteration of DNA methylation in plants as a response to experimental factors. The methylation changes were silicon dose-dependent. These modifications may suggest a mechanism for plant adaptation under salt stress after silicon application.

## 1. Introduction

The changing climate negatively affects the productivity of global agriculture, which poses a global threat to food security. The negative effects of environmental factors involve disruption of growth and development of crop plants. Major environmental stresses, such as high salinity, drought, cold, and heat have a damaging impact on plant survival and biomass production. As a result, there is a decrease in the yields of basic food crops by up to 70%. The adverse effect of excess minerals, such as Na^+^ and Cl^−^, on plants is called salt stress [1,2,3].

Saline soils are difficult to remediate due to the mobility of Na and Cl ions in the soil, and their spatial variability, which is the effect of interactions between the soil, the agronomic and the climatic environment [4]. More than 6% of the world’s surface is affected by salinization, and the problem of saline soil is growing constantly. The high concentration of salt in soil is estimated to cause the exclusion of approximately 1.5 million hectares of soil from production and reduces the potential for agricultural production by up to 46 million hectares per year [5]. The presence of salt in the soil solution reduces the ability of the plant to absorb water and causes osmotic, ionic and oxidative stress [1,6,7,8,9,10,11]. Excess salinity affects all cellular processes, including disruption of cellular homeostasis, impairment of photosynthesis, mRNA processing, transcription, protein synthesis, disruption of energy metabolism, amino acid biosynthesis, and lipid metabolism [12]. Under saline conditions, decreases in CO_2_ assimilation in the Calvin cycle are accompanied by a decrease in the photochemical electron sink, which in the presence of light affects the functioning and efficiency of photosystems [12,13]. In plants under salt stress conditions, osmotic shock occurs. The stomata close, which limits the ability to photosynthesize by reducing the supply of CO_2_ [7,12].

Currently, emphasis is placed on finding methods to reduce crop losses caused by various types of stress. One way is to use growth stimulants based on microelements in the form of spraying the above-ground organs of plants. Among the advantages of foliar fertilization, it is worth mentioning that it is a cheaper and more convenient method to use than soil fertilization. Moreover, the use of this type of application does not interfere with the soil environment and allows for quick delivery of nutrients to plants, which can be difficult, e.g., due to soil drought [14,15]. Furthermore, the use of salinity-tolerant cultivars is believed to be the most effective way to combat soil and water salinity [7]. Wheat (*Triticum aestivum* L.) is grown worldwide and currently constitutes a significant basis for human nutrition, and the demand for grain will increase in the coming years [16,17,18]. Therefore, it is important to look for opportunities to improve the yield of this species under stressful conditions. Silicon (Si) is a trace element that has been recognized as beneficial by the International Institute of Plant Nutrition because of its wide range of positive effects on plants. This element is not essential for the survival of most plant species, but numerous studies indicate that it increases stress tolerance and improves crop productivity [19]. Silicon is the second most abundant element on Earth after oxygen. The soil contains from 1 to 5% silicon in its active form. The form available to plants as a nutrient is mono-silicic acid (H_4_SiO_4_), and the amorphous form of silicon dioxide (SiO_2_) can also be easily taken up by plants. It is believed that the uptake of Si by cereals is higher than in other species [11]. The positive effects of Si in plants are related to the formation of the barrier of the plant cell wall, the regulation of antioxidant enzymes, and participation in the nutrient uptake mechanisms, especially under conditions of osmotic stress. Silicon fertilization can be an agrotechnical treatment that limits the impact of environmental stress and the depletion of nutrients in the soil. Consequently, it can be an alternative to maintaining sustainable agriculture [20]. Numerous studies report a beneficial effect of foliar and soil application of silicon on the yield of crop plants. The use of silicon in different doses improves morpho-physiological parameters and causes changes in the level of plant DNA methylation [21,22,23,24,25,26,27]. Photosynthesis converts solar energy into chemical energy, which is the energy source of almost all living things on Earth. Photosynthetic machinery consists of various mechanisms, including gas exchange systems, photosynthetic pigments, photosystems, electron transport systems, carbon reduction pathways, and enzyme systems [24]. Leaves are the main photosynthetic organs of plants. Leaf chlorophyll is an important indicator of nutrient stress, photosynthetic capacity, and plant growth status. Chlorophyll content plays an important role in understanding how photosynthesis functions in plants. Chlorophyll is an important part of the Calvin–Benson cycle and is responsible for light harvesting during photosynthesis, resulting in the excitation of electrons that are used to drive the production of nicotinamide adenine dinucleotide phosphate and chemical energy in the form of adenosine triphosphate [28,29,30,31,32]. Chlorophyll meters have been successfully used to estimate the chlorophyll content in the leaves of various plant species in a nondestructive manner, and particularly to study plant stress physiology [32,33]. Chlorophyll fluorescence, in addition to biochemical methods and gas exchange, is considered a reliable noninvasive technique for evaluating electron transport and related photosynthetic processes [34].

Epigenetic regulations are essential for the growth, development, and reproduction of plants and also play a key role in the generation or acquisition of resistance to various types of environmental stress factors [35,36,37,38]. The basis of plant resistance is the ability to regulate gene expression and protein function in a different way [36]. This ability enables epigenetic modification. Epigenetic modifications are changes in gene expression that occur without a change in DNA sequence. It is one of the molecular mechanisms by which plants can silence or super-activate selected DNA templates [38,39]. Epigenetic mechanisms regulation of gene expression can be carried out by DNA methylation, histone modifications, and by non-coding small RNA and chromatin remodeling [35,40]. More important is the fact that some of these changes are inherited by the next generations as part of epigenetic memory [35,41]. DNA methylation involves the addition of a methyl group (-CH_3_) to cytosine pyrimidine ring (5-methylcytosine, 5 mC or N6-methyldeoxyadenosine, 6 mA) [42,43], and generally occurs at CpG dinucleotides in a symmetric fashion. In this situation, cytosine (C) residue on one CpG is methylated, and the corresponding residue on the complementary strand will also be methylated. This pattern temporarily breaks down during DNA replication, when the unmethylated daughter (nascent) strand and the methylated parent strand create an asymmetrically methylated CpG dyad termed hemi-methylated DNA [43]. The methylation pattern is primarily sustained during DNA replication and cell division by DNA methyltransferases, including maintenance and de novo methylases. As a result, it is possible to faithfully transmit DNA methylation pattern to subsequent generations, which ensures transgenerational epigenetic inheritance [44,45,46].

Due to the need to expand knowledge on the possibility of increasing the resistance of crops to stress, studies were conducted to investigate the response of wheat seedlings to exogenous silicon application under salinity conditions. The research hypothesis was adopted, which assumes a positive effect of the application of foliar silicon on the physiological parameters of wheat seedlings.

## 2. Results

### 2.1. The Influence of Salt Stress and Foliar Si Supply on Chlorophyll Content Index

**Figure 1:**
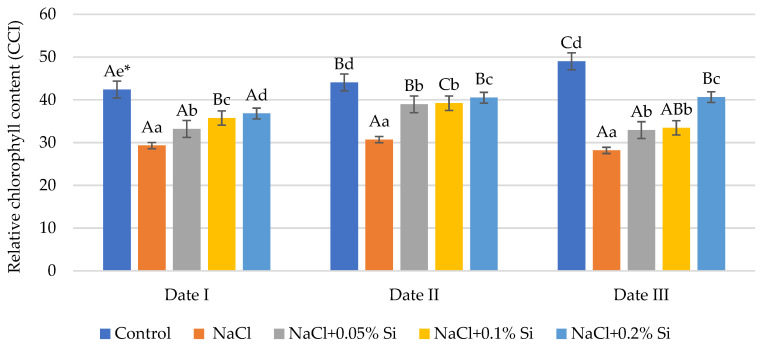
The impact of NaCl, concentrations of Si, and measurement date on chlorophyll content index (CCI) in wheat seedlings (Date I, Date II, Date III—7, 14, 21 days after Si application); data are as mean ± SD values. * Capital letters represent significant differences between means in subsequent measurement dates; lower case letters indicate differences between variants in a particular measurement date (*p* ≤ 0.05).

In the studies carried out, after NaCl application on each measurement date, a decrease in CCI was demonstrated compared to the control (on average 19.1% Date I, 13.2% Date II, and 31.3% Date III) (Figure 1). Exogenous Si application mitigated the negative effects of salt on all of the measured dates. On the first and second dates (Date I and Date II), each applied dose of foliar application caused a significant increase in CCI. The greatest increase was demonstrated after the application of the 0.2% Si dose (Date I—25.5% and Date II—31.3%) compared to plants of the NaCl variant. On the third measurement date (Date III), after applying the 0.2% Si dose, the increase in CCI was lower and amounted to 17.2%. A positive effect of silicon was also demonstrated with Si applications at doses of 0.1 and 0.2%. In process of time, an increase in the chlorophyll content was demonstrated in the control plants (Figure 1). On the second measurement date, plants treated with Si showed higher CCI values compared to the results obtained on the first date (Date I). On the third date (Date III), CCI decreased, except for the 0.2% silicon dose. This relationship indicates a positive effect of Si on the accumulation of CCI in wheat seedlings, especially in the first period after spraying.

### 2.2. The Influence of Salt Stress and Foliar Si Supply on Gas Exchange

**Figure 2:**
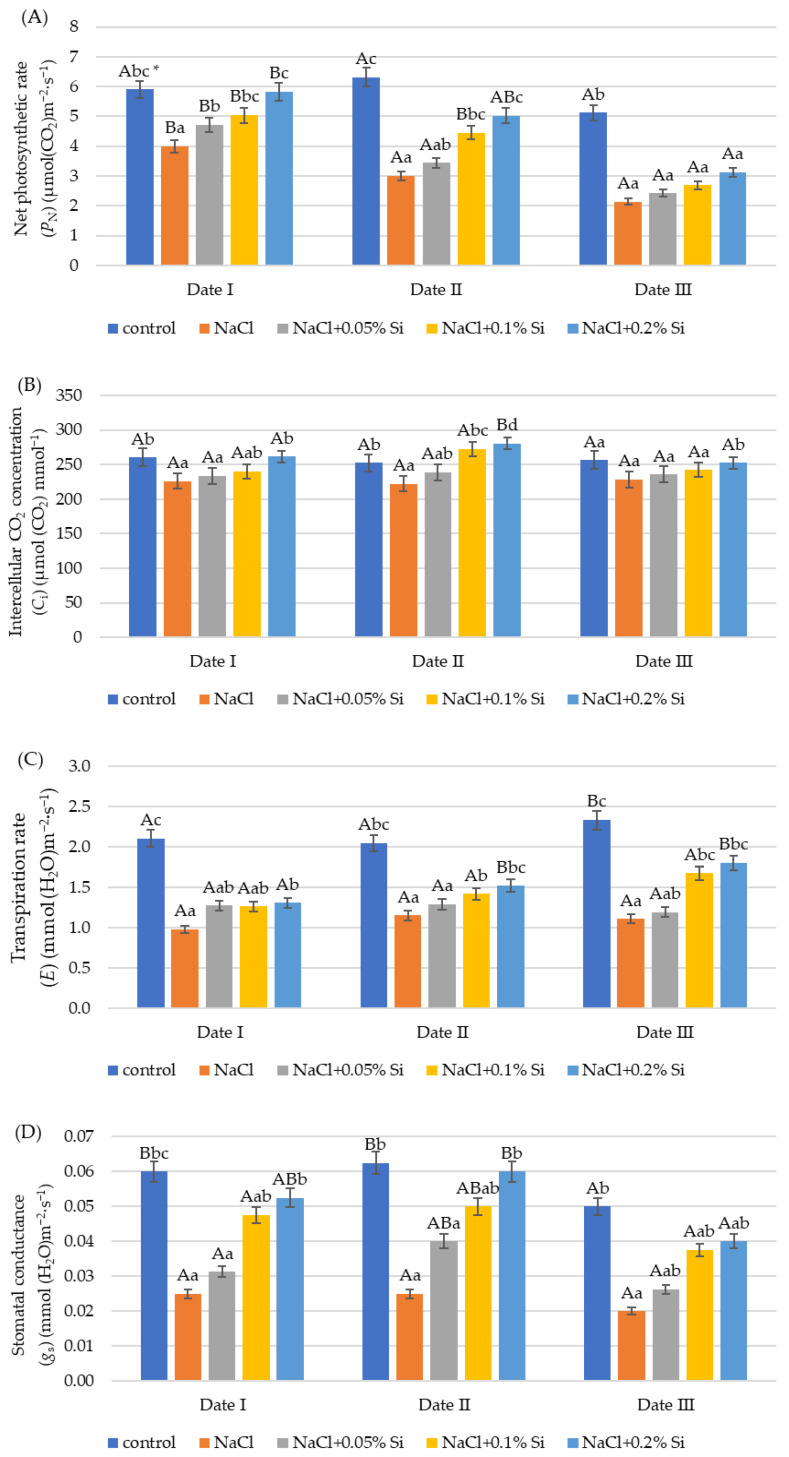
The impact of NaCl, concentrations of Si, and measurement date on gas exchange parameters: net photosynthetic rate (*P*_N_) (**A**) intercellular CO_2_ concentration (*C*_i_) (**B**), transpiration rate (*E*) (**C**), and stomatal conductance (*g*_s_) (**D**) in wheat seedlings (Date I, Date II, Date III—7, 14, 21 days after Si application); data are as mean ± SD values. * Capital letters represent significant differences between means in subsequent measurement dates; lower case letters indicate differences between variants in a particular measurement date (*p* ≤ 0.05).

In the experiment conducted, all the gas exchange parameters of the plants (*P*_N_, *E*, *C*_i_, *g*_s_) were highest in the control. The application of NaCl without addition of Si caused a significant decrease in the values of the analyzed indices (Figure 2). The largest decrease was observed compared to the control in the values of *g*_s_ and *E* (Figure 2C,D). Analysis of variance indicated a beneficial effect of the foliar application of silicon on the parameters analyzed. On each measurement date, the application of silicon in the amount of 0.2% Si increased the values of the analyzed parameters compared to plants without Si application and with soil salinity. A positive effect was observed in all the analyzed indices, such as *P*_N_, *C*_i_, *E*, and *g*_s_ (except for the measurement of *g*_s_ on Date III) (Figure 2). On Date I, an increase in the *P*_N_ was shown after the application of 0.1% and 0.2% Si doses (18.2% and 26.6%, respectively) compared to the NaCl variant. On Date II, a significant and positive effect of Si was observed at the dose of 0.2% Si, an increase in *P*_N_ (by 12.5%) and *C*_i_ (by 22.7%) (Figure 2A,B). On the third measurement date (Date III), the effect of silicon was smallest. A positive effect was shown only in the case of *C*_i_ after 0.2% Si application (an increase of 10.6%, respectively, compared to plants without application and grown in saline conditions) (Figure 2B).With the passage of measurement time, the values of the *P*_N_ index decreased. On Date III, a decrease in the *P*_N_ value was observed in all plants exposed to salt and the foliar application of silicon, compared to Date I (Figure 2A). Analyzing the *E* and *C*_i_ indices, on Date II, a slight increase was observed, *E* (20.4%) and *C*_i_ (7.2%), compared to Date I (Figure 2B,C). Only for the silicon dose of 0.2% was a significant increase observed. The *g*_s_ value after the application of 0.2% Si was significantly lower at the third measurement date. The parameter decreased by 20% and 33.4% compared to Dates I and II, which was shown by the analysis of variance (Figure 2D).

### 2.3. The Influence of Salt Stress and Foliar Si Supply on Chlorophyll Fluorescence

**Figure 3:**
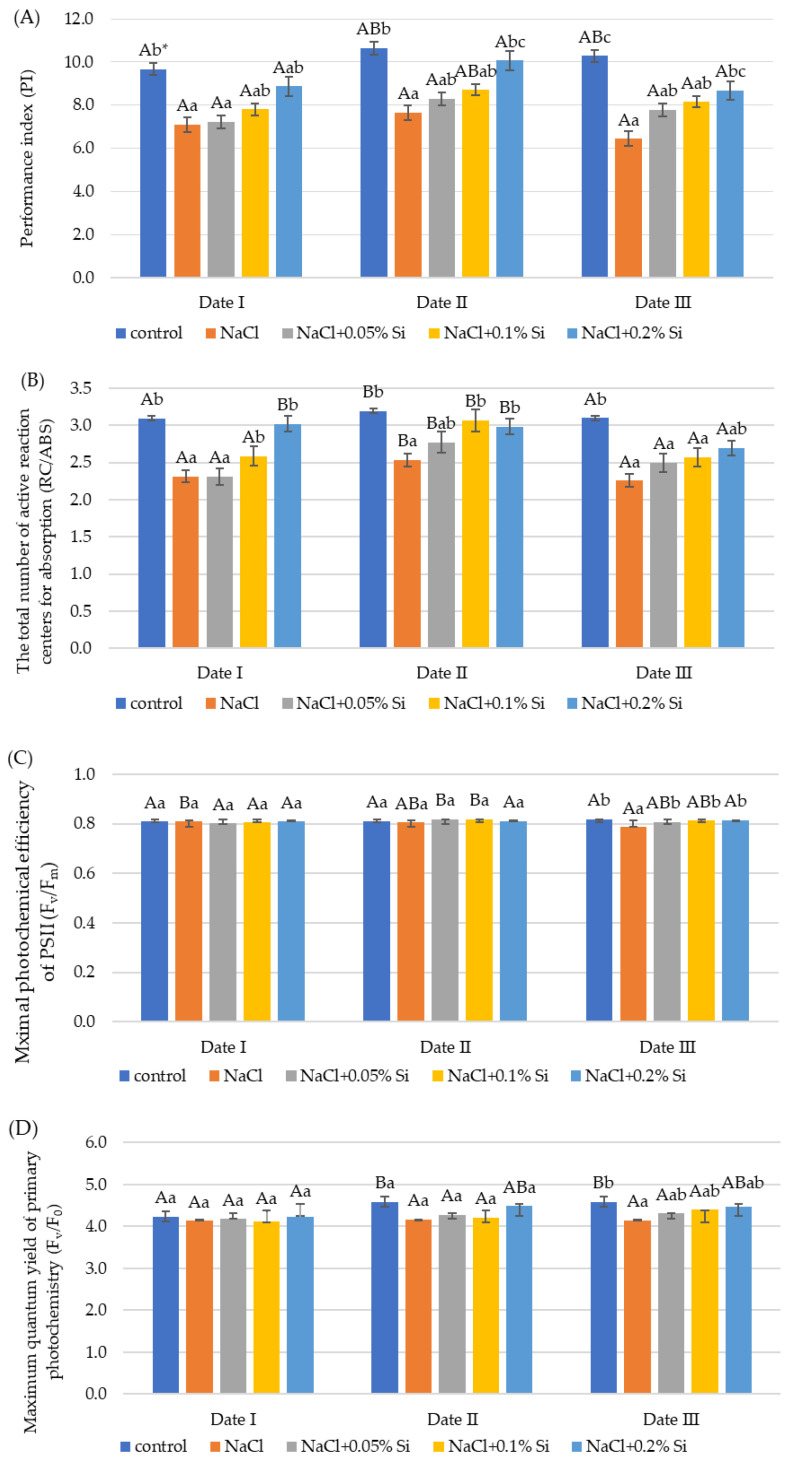
The impact of NaCl, concentrations of Si, and measurement date on chlorophyll fluorescence parameters: performance index (PI) (**A**), total number of active reaction centers for absorption (RC/ABS) (**B**), maximal quantum yield of PSII photochemistry (F_v_/F_m_) (**C**), and maximum primary photochemistry yield (F_v_/F_0_) (**D**) in in wheat seedlings (Date I, Date II, Date III—7, 14, 21 days after Si application); data are as mean ± SD values. * Capital letters represent significant differences between means in subsequent measurement dates; lower case letters indicate differences between variants in a particular measurement date (*p* ≤ 0.05).

Salt stress had a deleterious effect on chlorophyll fluorescence parameters (PI, RC/ABS, F_v_/F_m_, F_v_/F_0_) (Figure 3). The negative influence of salt was observed primarily in the PI values and the RC/ABS values. In each measurement term, in plants subjected to salt stress without silicon application, the PI and RC/ABS values were significantly reduced. The greatest decrease in values, 37.3% (PI) and 7.4% (RC/ABS), compared to the control was observed on Date III (Figure 3A,B). On this date, a decrease in F_v_/F_m_ by 4% was also noted (Figure 3C). It was shown that the foliar application of silicon had a positive effect on the chlorophyll fluorescence indices. The PI obtained higher values after applying each silicon dose compared to plants of the NaCl variant, but a statistically significant increase (26.0% and 25.6%, respectively) was confirmed at the 0.2% Si dose on the second and third measurement dates. On Date I, the foliar application of Si at levels of 0.1 and 0.2% caused a significant increase in RC/ABS by 24.9% and 31.5% compared to plants treated only with salt. On the second measurement date, the increase was 20.8% and 17.3%, respectively (Figure 3B). On the third measurement date, a positive effect of the exogenous application of Si was noted only at the highest dose. The F_v_/F_m_ indices and F_v_/F_0_ on the third measurement date increased after the application of each dose of Si, which was not observed on the previous dates. In plants in the NaCl variant, a significant decrease in F_v_/F_m_ was observed with time (Figure 3C,D). The variance analysis showed higher values of the RC/ABS parameter at Date II compared to Date I and Date III. An increase in the F_v_/F_0_ index was observed in the second and third dates only in control plants (Figure 3).

### 2.4. The Influence of Salt Stress and Foliar Si Supply on the DNA Methylation Level

The molecular analysis using MSAP markers indicated different levels of methylation among a group of analyzed plants. The results obtained showed a clear change in DNA methylation in wheat seedlings in response to the experimental factors used. The methylation changes were silicon dose-dependent. Using 5 combinations of primers led to obtained DNA products in a range of 217–231. The total methylation percentage was about 30% in all of the analyzed groups of plants, whereas the predominance of DNA hemi-methylation over symmetric methylation was visible in control as well as in salt-treated plants with or without silicon application (Table 1).

The results of the level of symmetric methylation were similar for most of the analyzed wheat groups and amounted to about 12.5–13.0%. The exception was for plants subjected to NaCl stress and treated with 0.05% silicon spray. For this group, the symmetric methylation was the lowest (10.8%). In the case of hemi-methylation, greater differences among groups of analyzed plants were visible (Table 1). Plants subjected to salt stress showed a higher level of hemi-methylation compared to control. However, silicon foliar application influenced the modification of the level of hemi-methylation. The observed differences were dependent on the dose used. The highest level of hemi-methylation was characterized by wheat plants sprayed with a 0.05% Si dose. The lower level of hemi-methylation indicated occurred in plants treated 0.2% Si, whereas, plants sprayed with a 0.1% Si dose indicated a moderate value of hemi-methylation (Table 1). However, in the case of the 0.05% Si dose, hemi-methylation exceeded the values shown in both the control group and the group subjected to NaCl stress. In turn, the group treated with the highest dose of Si showed the lowest hemi-methylation values (lower than the control group and the group treated only with NaCl). In the general assessment/calculation of total methylation, the differentiation of the overall methylation level was visible also. The highest values were shown in plants subjected to NaCl stress (33.6%), while the lowest was in plants sprayed with 0.2% Si. In the remaining groups tested and the control group, the level of total methylation was similar and amounted to about 32% (Table 1).

## 3. Discussion

Many studies indicate that salt stress causes negative effects in plants, including physiological and biochemical changes. These are most often manifested by a decrease in plant biomass and yields. The effects of stress depend on plant species and cultivars [12,47,48,49,50]. Among various field crops, including cereals, wheat is one of the most sensitive species to salinity, which reduces crop productivity and, at extreme concentrations, even causes complete yield loss [51]. In this study with wheat, we observed changes in plant physiology under soil salinity conditions at a level of 200 mM NaCl. We noted a significant decrease in the CCI after the application of salt at each measurement date. A particularly visible difference was observed between the NaCl variant and the control at Date III. A similar reduction in chlorophyll content under salt stress has been reported in many studies, in which the authors indicate that chlorophyll concentration is used as a sensitive indicator of the metabolic state of the cell [52,53,54,55]. The relationship between CCI and chlorophyll was found to be linear in the case of wheat. Studies show that using changes in CCI values provides diagnostic results between simulated and observed crop biomass [56]. In the study by Saddiq et al. [49], the effect of salinity at 0, 100 and 200 mM on *Triticum aestivum* L. genotype seedlings was assessed and the CCI in all genotypes decreased with increasing salt stress level. Similarly, in the studies by Akhtar et al. [56] and Loudari et al. [57], a decrease was reported in CCI in wheat under high NaCl conditions on the substrate. Salinity stress alters the size and number of chloroplasts, the accumulation of starch and lipids, and the organization of the lamellar membrane and disrupts transport across the cell membrane [12,58]. The total chlorophyll content in wheat plants was also significantly reduced by imposed salinity stress in the research by Shah et al. [59]. Zhu et al. [60] investigated the ultrastructure of control wheat chloroplasts stressed with 200 mM NaCl salt using transmission electron microscopy. Under salt stress, the chloroplasts had a slender spindle shape with loosely arranged granum thylakoids. Salinity stress reduces the synthesis of chlorophylls a, b, and carotenoids and also destroys protein bonds, which can lead to a decrease in the light absorption potential of photosynthetic pigments [61]. The negative impact of salt can be alleviated by applying silicon in the foliar application [62,63,64]. In this pot experiment, the foliar application of silicon fertilizer increased the CCI value compared to plants of the NaCl variant. This relationship was demonstrated in the case of faba beans, cotton, and other plants. In the faba bean, the use of silicon in the form of a foliar spray significantly improved the content of photosynthetic pigments, which were grown both under normal and saline conditions. The best effect was observed after the application of the highest concentration (1000 ppm SiO_2_) [23]. Thorne et al. [65] showed that the increase in chlorophyll content was a particularly noticeable result of adding Si to plants subjected to salt stress. The accumulation of chlorophyll varied in species and varieties under the influence of Si. In the study by Nisar et al. [66], foliar application of Si had a positive influence on chlorophyll content in cotton plants under saline conditions and the effect was dependent on genotype. The use of Si increases the content of photosynthetic pigments by reducing Na^+^ toxicity and maintaining the proper function and structure of chloroplasts [67,68,69]. Tuna et al. [70] showed that the chlorophyll content was significantly reduced in wheat cultivars grown under salt stress at 100 mM NaCl, while the application of Si completely restored the chlorophyll content to the level of control plants.

Among the various physiological traits, stomatal conductance and chlorophyll content are of the greatest importance in the search for crops resistant to high salinity levels. It is generally known that salt stress causes a marked reduction in stomatal conductance [7,71]. Photosynthesis depends on the exchange of CO_2_ between the atmosphere and the plant through the stomata. In addition to stomata, the photosynthetic CO_2_ pathway also results from the transport of CO_2_ from the mesophyll conductance to the carboxylation sites in the chloroplast stroma, where RuBisCo occurs to convert CO_2_ into the end product of photosynthesis. Gaseous exchange in the plant is impaired when the water potential of the leaves decreases. Plants under salinity stress also accumulate wax in the epidermis to overcome transpiration losses, reducing the gaseous exchange attributes, and thus the photosynthetic process is limited [58].

In this study, a significant decrease in all measured gas exchange parameters was reported. Salinity stress also reduced *P*_N_ and *g*_s_ in wild-type wheat plants in the study of Zuo et al. [72]. Khoshbakht et al. [73] investigated the effects of salinity on gas exchange and physiological traits of nine citrus species. Gas exchange (*P*_N_, *g*_s_, *C*_i_) was negatively affected by salinity. Photosynthesis, stomatal conductance and photosynthetic pigment concentration were substantially decreased in maize at 68 and 102 mM NaCl in the study by Hichem et al. [74], and reduction in photosynthesis was mainly caused by closure of the stomata and partly by PSII photoinhibition. The functioning of photosystem II (PS II) shows high sensitivity to salt stress [75]. Salt stress causes a decrease in the rate of CO_2_ assimilation by reducing both stomatal and root conductance, leading to an excessive reduction of PSII, along with electron diversion to molecular oxygen that generates reactive oxygen species (ROS). ROS are considered to be the main source of structural damage under abiotic stress conditions. ROS are highly cytotoxic and their excess can severely disrupt metabolic pathways [12,49,58,65,76,77]. Reduced photosynthetic efficiency is considered to be the major salt-induced limitation that inhibits plant growth and ultimately crop productivity. In the experiment conducted, an increase in gas exchange parameters was observed after foliar application of Si. This relationship was demonstrated for all plant gas exchange indices, especially for the NaCl^+^ 0.2% Si variant. An increase in *P*_N_, *g*_s_ and *E* was observed after Si supplementation in sorghum plants subjected to salt stress at a level of 100 mM NaCl [78]. The mitigating role of Si involves reducing Na^+^ and Cl^−^ uptake, increasing mineral nutrition and biosynthesis of various dissolved substances, reducing oxidative stress, modifying gas exchange attributes, and stimulating gene expression [58,62,66,79]. Si may affect stomatal function by increasing their conductance, and therefore the rate of photosynthesis, which may reduce transpiration and thus limit water loss [65]. Many authors report that silicon can improve ROS removal by influencing the antioxidant content of plants, and this effect varies depending on the plant species [79,80,81].

Chlorophyll fluorescence measurement is a useful indicator for quantifying salt-induced damage to the photosynthetic apparatus [82]. This test was useful in assessing wheat photosynthetic responses to abiotic stresses [83]. Leaf CO_2_ assimilation, PSII efficiency, and their relationship allow the use of fluorescence to assess genotype resistance [49]. Photosystem II (PSII) has been identified as the main component of the photosynthetic system sensitive to salt stress. Salinity causes a reduction in key chlorophyll fluorescence parameters, such as F_v_/F_m_ and PI [81]. In our study, we observed a significant reduction in PI and RC/ABS in plants from the NaCl variant at all measurement dates and F_v_/F_m_ and F_v_/F_0_ at Date III compared to the control. The reduced ratio F_v_/F_m_ under salinity stress in wheat was also reported in the study by Yadav et al. [84]. Hichem et al. [74] reported that salinity stress caused a decrease in the parameter F_v_/F_m_ in maize, and the observed effect was dependent on the variety. Reduced photosynthetic efficiency under salinity stress conditions can be attributed to biochemical limitations, such as impaired RuBP regeneration, chlorophyll degradation, and chloroplast ATP synthesis [58]. In the conducted research, the best effect of exogenous Si application was observed after the application of the highest dose—0.2%. The positive effect of silicon under stress conditions by improving the F_v_/F_m_, and F_v_/F_0_ values was observed in many plant species [85]. Silicon partially reduced the negative effect of NaCl stress, increasing the tolerance of tomato plants to NaCl salinity by increasing the photochemical efficiency of PSII, superoxide dismutase (SOD) and catalase (CAT) activity, chlorophyll content, and PSII photochemical efficiency [25].

One of the epigenetic mechanisms that regulate genome functioning and induce plant resistance and adaptation to abiotic stresses in plant’s responses to environmental stresses is DNA methylation [86,87]. The gene body methylation may have an important role in regulating gene expression in an organ- and genotype-specific manner under stress conditions [88]. Molecular mechanisms of plant response to stress are based on the adjustment of gene expression towards, for example, the production of secondary metabolites that influence the development of signals necessary for the activation of defense mechanisms in stress conditions [89]. Modification of gene expression seems to be, therefore, the first stage of plant adaptation to stress conditions leading to the ‘coping of plants in stress conditions’. Environmental signals, like salt stress, have been shown to induce epigenetic mechanisms, like DNA methylation and histone modifications, ultimately leading to plant adaptation [35,90]. Analyses performed on numerous plants, like cotton [91], *J. curcas* [92], and maize, as well as cereals like wheat [87] or barley [93], led to confirmation of the changes in DNA methylation pattern in reaction to stress conditions. The role of epigenetic modifications in plants’ response to stress has also been researched by comparison of the methylation level in plants resistant and sensitive to a specific stress factor. Zhong et al. [39] showed that CCGG sequences of salt-tolerant wheat plants (Dekang-961 plants) were more methylated than those of salt-sensitive wheat plants (Lumai-15). The scientific reports above indicate that it is reasonable to analyze the level of methylation in the context of plant response and resistance to stress conditions. In the presented research, differences in methylation level were performed in wheat plants treated and untreated with salt stress induced by NaCl. Additionally, the stressed plants treated with silicon differ in percentages of methylation from each other, as well as between control or NaCl-only treated plants. The difference was silicon dose-dependent. Particularly, a visible difference was demonstrated, in the case of hemi-methylation, considered as methylation occurring during the response of plants to stress. Analysis of the white poplar *Populus alba* L. DNA methylation profiles showed that environmental conditions strongly influence internal cytosine hemi-methylation [94]. This suggests that the application of silicon during salt stress generated modification of gene expression in the direction of plants’ ability to ‘cope’ with stress conditions. An effect of this phenomenon is the modification of the physiological and biochemical reactions of wheat plants on stress, presented in the above research.

## 4. Materials and Methods

### 4.1. Plant Growth Conditions

At the University of Rzeszów (Poland), research was conducted under controlled conditions. In pots with a diameter of 15 cm, in which 1.5 kg of soil with a grain size typical of loamy sand and a slightly acidic (pH = 5.9) were placed, wheat seeds (10 pieces per pot) of Bogatka cultivar (DANKO Hodowla Roślin Ltd., Choryń, Poland) were sown. The level of macro-elements in soil (mg·100 g^−1^) was as follows: P_2_O_5_ 17.1, K_2_O 17.0, Mg 8.87, Ca 9.46. The pot experiment was set up in a growth chamber (model GC-300/1000, JEIO Tech Co., Ltd., Seoul, Republic of Korea). The plants were grown for a 14 h day length and a day/night temperature cycle of 21 °C/18 °C. To induce salinity stress in the first leaf pair stage (BBCH 12) [95], commercial salt (NaCl) was added twice a day for two days at an amount of 50 mM to obtain a concentration of 200 mM and to avoid osmotic shock. After 7 days from the application of NaCl solution, silicon (Si) fertilizer Optysil (content 200 g·L^−1^ SiO_2_) (Intermag Ltd., Olkusz, Poland) was applied foliarly. The preparation was used in three concentrations: 0.05, 0.1 and 0.2%. The control consisted of plants in pots without the addition of NaCl and Si. The experimental design was a completely randomized factorial design with three replicates. The positions of the pots in the experiment were changed randomly every week. The spraying of Optysil was performed with a handheld laboratory sprayer (Venus Super HD solvent line, Kwazar Corporation Ltd., Jaktorów, Poland). A measurement of 3 mL of solution was used for each pot. Deionized water was added to the control variant. Physiological measurements of wheat seedlings were performed on the first or second fully expanded leaf; 7 (Date I), 14 (Date II) and 21 (Date III) days after silicon fertilizer application.

### 4.2. Chlorophyll Content Measurement

Chlorophyll content index (CCI) was measured using a portable, nondestructive, and lightweight instrument (CCM-200; Opti-Sciences Inc., Hudson, NH, USA). CCM-200 was adopted to collect CCI values from the first fully expanded functional leaf of each plant. A total of 10 plants were randomly measured in each plot.

### 4.3. Measurement of Gas Exchange Parameters

The following were measured on two fully developed leaves: net photosynthetic rate (*P*_N_), intercellular CO_2_ concentration (*C*_i_), transpiration rate (*E*) and stomatal conductance (*g*_s_). The LCpro-SD photosynthesis measurement system (ADC Bioscientific Ltd., Herts, UK) was used for measurements. The LCpro-SD leaf photosynthesis chamber has a flow accuracy of ±2%. The light intensity in the measuring chamber was 350 µmol·m^−2^·s^−1^, and the temperature was 23 ± 2 °C. Three measurements were taken in each pot.

### 4.4. Measurement of Chlorophyll Fluorescence Parameters

Chlorophyll fluorescence measurements, i.e., photosynthetic efficiency index (PI), total number of active reaction centers for absorption (RC/ABS), maximum quantum yield of PSII photochemistry (F_v_/F_m_), and maximum quantum yield of primary photochemistry (F_v_/F_0_), were performed with the Handy PEA fluorimeter (Hansatech Instruments, King’s Lynn, Norfolk, UK). Red actinic light (wavelength at peak 650 nm; spectral line 22 nm) with an intensity of 3500 μmol·m^−2^·s^−1^ was used for the induction of fluorescence and 1 s of transient fluorescence was recorded. Measurement was carried out in the middle of the leaf blades away from the main leaf vein after dark adaptation (30 min) using leaf clips. Five measurements were taken in each pot.

### 4.5. Determination of Methylation-Sensitive Amplification Polymorphism (MSAP)

Molecular analysis was performed with Methylation Sensitive Amplification Polymorphism markers (MSAP) by Xiong et al. [96] and Xu et al. [97] with modifications. For molecular analysis, the plant material, e.g., leaves, was collected at the end of experiments and frozen in liquid nitrogen at −80 °C. For all groups of plants, the bulk of leaves were subjected to DNA isolation according to protocol followed by Doyle and Doyle [98]. To perform MSAP, only DNA of high quality (absorbance by λ260/280 in range 1.8–2.0) was used. The MSAP analysis consisted of 4 reactions: (1) restriction, (2) ligation, (3) pre-amplification, and (4) selective amplification. For the restriction enzymes like EcoRI, MspI, and HpaII (Thermo Scientific, Waltham, MA, USA), were used. MspI and HpaII are isoschizomers that recognize the same DNA sequence 5′CCGG3′. The capacity to cleave at the recognized sequence by the MspI and HpaII is dependent on the methylation state of the external or internal cytosine residues. The HpaII is inactive if one or both cytosines are fully methylated (both strands methylated; symmetric methylation) but cleaves the hemi-methylated sequence (only one DNA strand methylated), whereas MspI cleaves methylation 5′CmCGG3′ on both strands of DNA but not 5′mCCGG3′ [96]. In the restriction reaction, 500 ng of genomic DNA was digested with EcoRI/MspI (Thermo Scientific, Waltham, MA, USA) and EcoRI/HpaII (Thermo Scientific, Waltham, MA USA) restriction enzymes for 2 h at 37 °C. The products of the restriction reaction were then subjected to ligation with EcoRI and MspI or HpaII-specific adapters (Genomed, Warsaw, Poland). Subsequently, the ligated DNA was diluted to 1:10 and pre-amplified using EcoRI (Genomed, Warsaw, Poland) and MspI or HpaII (Genomed, Warsaw, Poland) primers with one selective nucleotide at the 3′ ends. The pre-amplified product was diluted 10× with TE buffer and, subsequently, selectively amplified with different combinations of MSAP primers (Genomed, Warsaw, Poland) (Table 2).

The PCR reactions were performed in 30 cycles with the following profile: 94 °C for 120 s pre-denaturation, 30 s denaturation at 94 °C annealing for 30 s, and extension at 72 °C for 60 s, ending to complete extension for 5 min at 72 °C. In the case of pre-amplification, the temperature of annealing was 46 °C, whereas annealing for selective amplification was initiated at 65 °C, and then reduced by 0.7 °C for the 11 cycles. The next cycles (18) were performed with annealing at 56 °C for 30 s.

Selective amplification products were separated by electrophoresis on 6.0% denaturing polyacrylamide sequencing gel. The gel was pre-run for about 30 min before loading the probes and then ran for about 70 min at 60 W. Detection of obtained products was performed with the silver nitrate method [99].

### 4.6. Methylation Data Analysis

MSAP techniques were performed according to the methodology described by Xu et al. [97]. The MSAP bands’ gel template was transformed into a binary character matrix, using “0” and “1” to indicate the presence or absence of bands (PCR product) for particular loci. A symmetric methylation event was observed when bands present in the gel from the reaction EcoRI + MspI (M) were absent from the reaction EcoR I + HpaII (H). In this case, the internal cytosine of 5′CCGG3′ sequence was methylated (5′CmCGG 3′) (‘symmetric or fully methylation’ defined). Simultaneously, the simultaneous presence of a band in H and the absence in M indicated that the external cytosine of one DNA 5′CCGG3′ sequence strand was methylated (5′mCCGG3′). This is determined as the ‘hemi-methylated state’.

Percentage methylation was calculated according to a formula defined by Xiangqian et al. [100]:

Methylation (%) = (number of methylated bands)/(total number of bands) × 100(1)

### 4.7. Statistical Analysis

The results obtained were statistically analyzed. The package TIBCO Statistica 13.3.0 (TIBCO Software Inc., Palo Alto, CA, USA) was used. The Shapiro–Wilk test was performed at *p* = 0.05. Then, the homogeneity of variance was assessed and a two-way ANOVA with repeated measures was performed (the date of measurement was a factor of the experiment). Tukey’s post hoc test was also performed at a significance level of *p* ≤ 0.05 [101].

## 5. Conclusions

For wheat, the most beneficial dose of silicon under salt stress conditions was 0.2% Si. After its application, the highest CCI value was shown in leaves. Wheat plants also obtained higher gas exchange rates: *P*_N_, *C*_i_, *E*, *g*_s_ and chlorophyll fluorescence parameters: PI, RC/ABS F_v_/F_0_, F_v_/F_m_, compared to lower applied doses of Si—0.05 and 0.1%. The effect of silicon was more visible after application on the first and second measurement dates compared to that on the third date.

Analyses performed on salt stress-treated wheat indicated modifications in DNA methylation in reaction to stress conditions. Modification of methylation level as an effect of silicon application confirms its impact on plant response and resistance to stress conditions. A better understanding of epigenetic regulation of plant growth and response to environmental stresses may create novel heritable variations for crop improvement.

The results of the research indicate the need to expand research on the positive role of silicon in wheat. This is particularly important in the face of climate change and the intensification of abiotic stress. Future work should focus on examining the role of foliar application of Si in the production of wheat in soils where salinity is a problem. In addition, it is important to examine the interaction of the foliar application of Si and changing weather conditions, which can modify the effects of treatments.

## Figures and Tables

**Table 1:** Comparison of methylation level among analyzed groups of wheat.

Analysed Parameters:	Control	NaCl	NaCl + 0.05% Si	NaCl + 0.1% Si	NaCl + 0.2% Si
Number of symmetric methylation bands	29	28	25	28	28
Symmetric methylation (%)	13.1	12.9	10.8	12.5	12.5
Number of hemi-methylation bands	42	45	50	44	40
Hemi-methylation (%)	19.0	20.7	21.6	19.8	17.9
Total bands number	221	217	231	224	224
% Total methylation	32.1	33.6	32.5	32.1	30.4

**Table 2:** Sequences of primers and adapters used for reaction and stages of MSAP analysis.

MSAP Stage	Primer/Adapter	Sequence
Ligation	EcoRI-Adapter	5′CTCGTAGACTGCGTACC3′ 3′CATCTGACGCATGGTTAA5′
	MspI-HpaII-Adapter	5′CGACTCAGGACTCAT3′ 3′TGAGTCCTGAGTAGCAG5′
Preamplification	Pre-EcoRI	5′GACTGCGTACCAATTC3′
	Pre-MspI-HpaII	5′GATGAGTCCTGAGTCGG3′
Selective amplification	EcoRI-ACT × MspI/HpaII-CT	5′GACTGCGTACCAATTCACT3′ 5′GATGAGTCCTGAGTCGGCT3′
	EcoRI-AG × MspI/HpaII-CTC	5′GACTGCGTACCAATTCAG3′ 5′GATGAGTCCTGAGTCGGCTC3′
	EcoRI-AC × MspI/HpaII-ATG	5′GACTGCGTACCAATTCAC3′ 5′GATGAGTCCTGAGTCGGATG3′
	EcoRI-AT × MspI/HpaII-CTC	5′GACTGCGTACCAATTCAT3′ 5′GATGAGTCCTGAGTCGGCTC3′
	EcoRI-AT × MspI/HpaII-CAT	5′GACTGCGTACCAATTCAT3′ 5′GATGAGTCCTGAGTCGGCAT3′

## Data Availability

The data presented in this study are available upon reasonable request from the corresponding author.
